# Exercise order in school-based concurrent training for adolescents with obesity

**DOI:** 10.1016/j.isci.2026.116136

**Published:** 2026-07-06

**Authors:** Yuhang Gao, Chunyan Dai, Jiahui Ke, Xiaodong Wang, Ti Zhang, Meng Cao, Yan Xie

**Affiliations:** 1 Faculty of Health and Wellness, City University of Macau, Macao SAR, China; 2 School of Physical Education and Training, Xi’an Physical Education University, Xi’an 710068, China; 3 Sports College, Shenzhen University, Shenzhen 518060, China; 4 Wang Xiaodong Sports Rehabilitation Studio, Bao’an District, Shenzhen 518060, China; 5 Shenzhen Nanshan Center for Chronic Disease Control, Shenzhen 518060, China; 6 Shenzhen Futian Third People’s Hospital, Shenzhen 518000, China

**Keywords:** health sciences, obesity medicine

## Abstract

Adolescent obesity requires time-efficient school-based exercise strategies that improve fitness and body composition within routine physical education settings. This randomized controlled trial compared two 12-week exercise sequences combining repeated sprint exercise and bodyweight resistance exercise in adolescents with obesity: bodyweight resistance followed by repeated sprint exercise (CRS) and repeated sprint exercise followed by bodyweight resistance (CSR), alongside a non-exercising control group. Fifty-nine participants completed the trial. After adjustment for baseline values, both CRS and CSR showed more favorable post-intervention outcomes than the control group for VO_2_max, BMI, body fat percentage, waist circumference, 20-m shuttle run performance, sit-ups, rope skipping, fat-free mass, and resting heart rate. Most outcomes were similar between CRS and CSR, but CRS produced greater improvement in VO_2_max. These findings support flexible school-based combined high-intensity exercise programming, while suggesting that resistance-before-sprint sequencing may better target cardiorespiratory fitness.

**Trial registration:**

Chinese Clinical Trial Registry ChiCTR2500103429; ethics approval PN-202400005.

## Highlights

• School-based concurrent training improved fitness and adiposity outcomes
• Exercise order had limited effects on most health-related outcomes
• Resistance-before-sprint sequencing produced greater VO_2_max gains
• Flexible sequencing may support physical education implementation

## Graphical abstract

**Figure:**
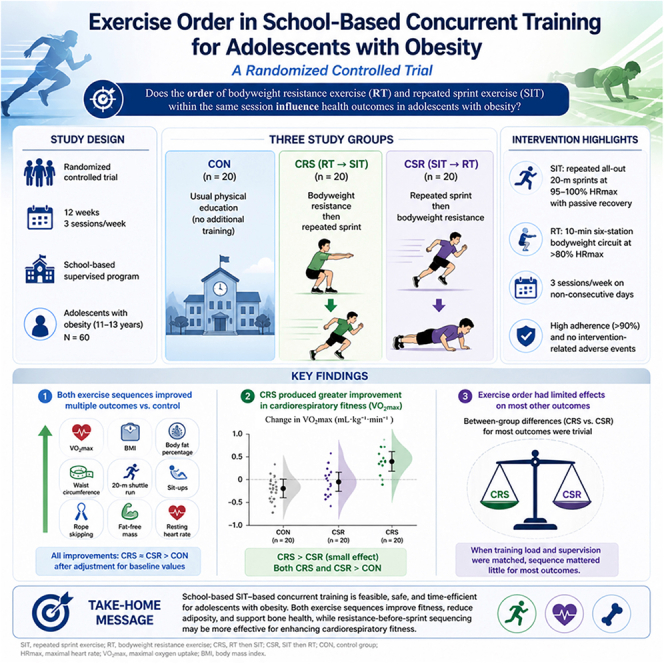

## Introduction

Childhood obesity is a major global health concern and is associated with early cardiometabolic abnormalities, including hypertension, dyslipidemia, and insulin resistance, which may persist into adulthood.^1^^,^^2^^,^^3^ Beyond these metabolic consequences, excess adiposity during youth has also been linked to impaired skeletal development, lower bone mineral density (BMD), reduced bone mineral content (BMC), and altered bone accrual during a critical period of growth.^4^^,^^5^^,^^6^ Given the long-term implications of adolescent obesity for future metabolic health and fracture risk, there is a clear need for interventions that can reduce adiposity while also supporting fitness and bone health in school-aged populations.^1^^,^^2^^,^^6^

Exercise is a cornerstone of pediatric obesity management.^7^^,^^8^ In school settings, however, the key issue is not only whether exercise is effective, but whether it can be delivered in a format that is practical, time-efficient, and feasible within routine physical education lessons.^9^^,^^10^^,^^11^ Current physical activity guidelines recommend that children and adolescents engage regularly in moderate-to-vigorous physical activity, including activities that strengthen muscle and bone.^12^ In practice, school-based programs are often constrained by limited lesson time, large class sizes, restricted equipment, and the need for simple, well-supervised activities.^9^^,^^10^ Under these conditions, brief high-intensity formats that combine repeated sprint exercise with bodyweight resistance exercise may offer a realistic and scalable strategy for improving health-related outcomes in adolescents with obesity.^9^^,^^10^^,^^11^^,^^13^

Previous school-based studies have shown that high-intensity interval-type exercise and combined exercise programs can improve cardiorespiratory fitness and body composition in youth with overweight or obesity.^10^^,^^11^^,^^14^ More recent interventions using repeated sprint efforts together with bodyweight circuit-based exercise have also reported favorable effects in real-world school environments.^15^^,^^16^^,^^17^ However, evidence is limited regarding whether the within-session order of these two components influences adaptation.^18^^,^^19^ This issue is relevant not only from a physiological perspective, but also from a practical one, because in actual physical education classes teachers may need to rotate students between sprint-based and calisthenic stations according to space, class organization, and supervision demands.

Although some previous studies have discussed sequence effects within broader combined-training models, the present intervention differs from traditional laboratory-based aerobic-plus-resistance programs.^19^^,^^20^ In the current study, the aerobic component consisted of repeated short sprint efforts, whereas the resistance component involved bodyweight calisthenics delivered in a school setting. Accordingly, the present trial was designed primarily to examine the order of two school-feasible high-intensity exercise components within the same lesson, rather than to test a classical interference-effect model. Existing evidence on this question remains limited and mixed, with some studies suggesting small order-dependent differences and others reporting broadly similar responses when total training dose is matched.^18^^,^^19^^,^^21^

Therefore, the present randomized controlled trial compared two school-based exercise sequences in adolescents with obesity: bodyweight resistance exercise followed by repeated sprint exercise (CRS) and repeated sprint exercise followed by bodyweight resistance exercise (CSR), together with a non-exercising control group (CON). The primary outcome was VO_2_max, and secondary outcomes included body mass index (BMI)-z, body composition, bone-related variables, and additional fitness and physiological measures. We hypothesized that both exercise sequences would lead to more favorable changes than CON, whereas differences between CRS and CSR would be limited for most outcomes, although VO_2_max might still show some sensitivity to exercise order.

## Results

### Participant flow, retention, and adherence

**Figure 1:**
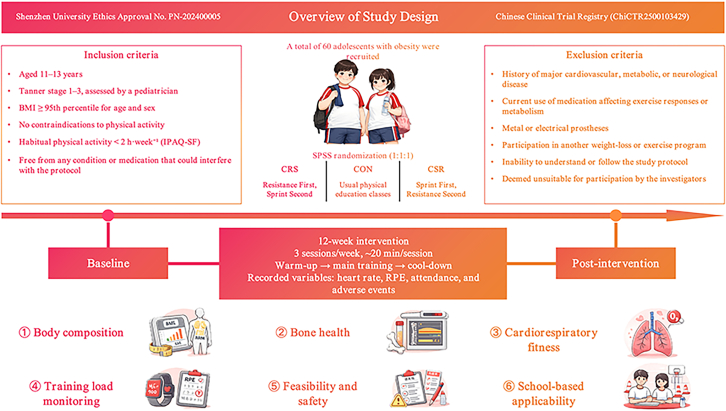
Study design and timeline of the 12-week school-based exercise trial The figure summarizes baseline assessment, randomization, the 12-week intervention period, post-intervention assessment, and the main outcome domains, including cardiorespiratory fitness, body composition, bone-related variables, functional fitness, and physiological measures.

**Figure 2:**
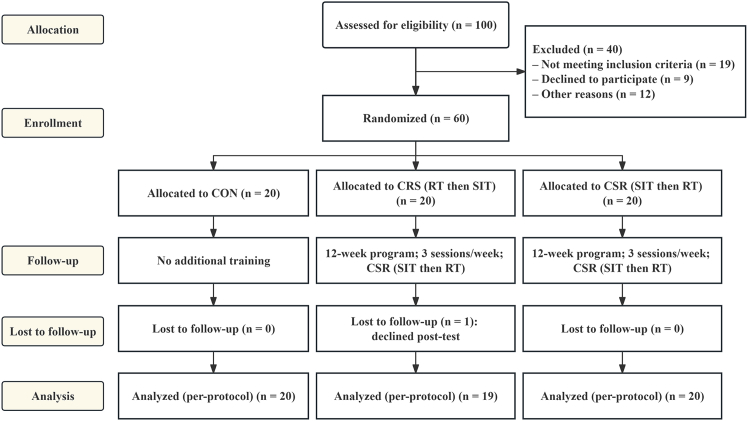
CONSORT flow diagram of participant progress through the trial The diagram shows participant screening, exclusion, randomization, allocation to the CON, CRS, and CSR groups, withdrawal during follow-up, and final inclusion in the analyses.

**Figure 3:**
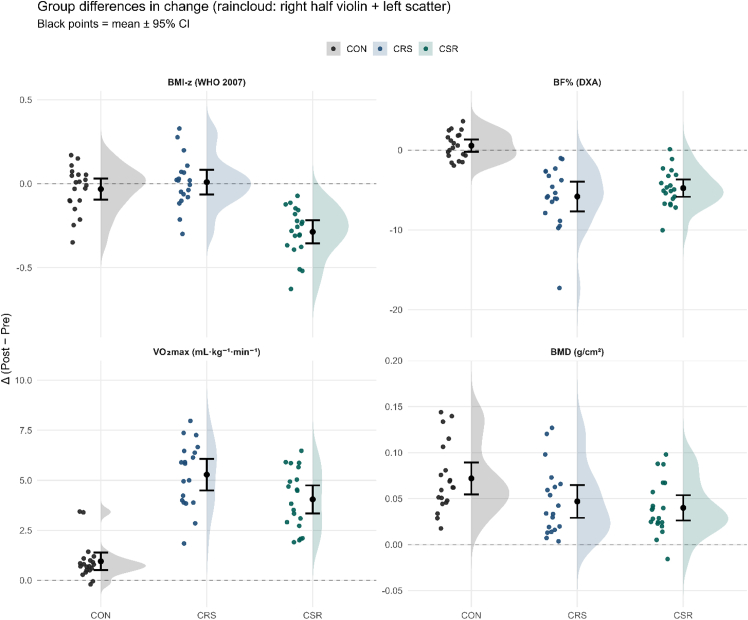
Group differences in change scores for selected primary and secondary outcomes Values represent change from baseline to post-intervention. Error bars indicate 95% confidence intervals. CON, control group; CRS, bodyweight resistance exercise followed by repeated sprint exercise; CSR, repeated sprint exercise followed by bodyweight resistance exercise.

A total of 100 adolescents with obesity were assessed for eligibility. Forty participants were excluded because they did not meet the inclusion criteria (*n* = 19), declined to participate (*n* = 9), or for other reasons (*n* = 12). The remaining 60 eligible participants were randomized equally to the control group (CON, *n* = 20), the resistance-then-sprint group (CRS, *n* = 20), and the sprint-then-resistance group (CSR, *n* = 20). During the 12-week intervention, one participant in the CRS group withdrew because they declined post-intervention testing (Figure 1). Consequently, 59 participants completed the study and were included in the per-protocol analyses (CON, *n* = 20; CRS, *n* = 19; CSR, *n* = 20). No serious adverse events were reported during the trial. Participant flow is shown in Figure 2, and baseline characteristics are summarized in Table 1. Outcome-specific ANCOVA results are presented in Table 2 and Figure 3.

**Table 1:** Baseline characteristics of participants by group

Variable	All (*n* = 59)	CON (*n* = 20)	CRS (*n* = 19)	CSR (*n* = 20)	*p*
Age (years)	12.4 ± 0.3	12.4 ± 0.3	12.3 ± 0.3	12.4 ± 0.4	0.949
Sex, boys/girls	47/12	16/4	15/4	16/4	0.996
Height (cm)	160.7 ± 7.6	160.8 ± 6.4	162.2 ± 9.3	159.1 ± 7.1	0.462
Weight (kg)	70.5 ± 10.7	70.2 ± 7.2	71.3 ± 13.2	67.4 ± 10.5	0.150
BMI (kg/m^2^)	27.2 ± 2.4	27.1 ± 0.9	28.0 ± 3.1	26.5 ± 2.5	0.133
BMI-for-age *Z* score	2.4 ± 0.4	2.5 ± 0.2	2.4 ± 0.5	2.4 ± 0.3	0.626
BF%	40.3 ± 7.3	40.8 ± 10.7	41.0 ± 3.7	39.0 ± 5.9	0.630
Waist circumference (cm)	91.4 ± 7.5	91.1 ± 7.3	94.1 ± 8.6	89.0 ± 5.9	0.110
WHR	0.9 ± 0.1	0.9 ± 0.1	1.0 ± 0.1	0.9 ± 0.0	0.153
Fat-free mass (g)	42.0 ± 7.4	41.4 ± 7.9	43.5 ± 7.3	41.1 ± 7.2	0.552
VO_2_max (mL·kg^−1^·min^−1^)	39.6 ± 3.8	40.8 ± 3.0	38.2 ± 4.1	39.7 ± 4.0	0.107
Resting HR (bpm)	76.9 ± 9.8	74.3 ± 9.4	79.6 ± 9.2	77.0 ± 10.6	0.241
SBP (mmHg)	117.3 ± 8.7	117.0 ± 6.9	117.9 ± 8.1	116.8 ± 11.1	0.919
DBP (mmHg)	68.5 ± 6.8	67.2 ± 6.7	68.3 ± 6.2	70.0 ± 7.3	0.426
BMD (g/cm^2^)	0.972 ± 0.086	0.973 ± 0.067	0.984 ± 0.097	0.960 ± 0.093	0.704
BMC (g)	1,932.9 ± 295.3	1,929.9 ± 306.4	1,958.7 ± 323.9	1,911.4 ± 267.8	0.885

Values are presented as mean ± SD unless otherwise indicated. Sex is presented as n/n (boys/girls). Between-group differences at baseline were assessed using one-way ANOVA for continuous variables and the chi-square test for categorical variables.

BMI, body mass index; BMI z-score, BMI-for-age *Z* score based on the WHO 2007 reference; BF%, body fat percentage; WHR, waist-to-hip ratio; VO_2_max, maximal oxygen uptake; HR, heart rate; SBP, systolic blood pressure; DBP, diastolic blood pressure; BMD, bone mineral density; BMC, bone mineral content.

**Table 2:** Effects of the intervention on body composition, fitness, bone, and physiological outcomes

Outcome	Group	Pre, mean ± SD	Post, mean ± SD	Change, mean difference (95% CI)	Adjusted post mean (95% CI)	Group effect F	*p*	Partial η^2^	Pairwise comparisons (adjusted *p*)
**Primary outcome: VO**_**2**_**max**									

VO_2_max	CON	40.80 ± 3.00	41.73 ± 3.00	0.95 (0.52, 1.38)	40.56 (39.92, 41.19)	45.88	<0.001	0.625	CRS vs. CON <0.001
	CRS	38.22 ± 4.06	43.51 ± 4.43	5.28 (4.49, 6.07)	44.84 (44.18, 45.49)				CSR vs. CON <0.001
	CSR	39.69 ± 4.02	43.73 ± 4.13	4.05 (3.35, 4.75)	43.63 (43.01, 44.26)				CRS vs. CSR = 0.030

**Body composition: BMI-z, BMI, BF%, weight, WC, WHR, FFM**									

BMI-z	CON	2.49 ± 0.23	2.46 ± 0.25	−0.032 (−0.095, 0.031)	2.40 (2.34, 2.47)	23.99	<0.001	0.466	CSR vs. CON <0.001
	CRS	2.44 ± 0.53	2.45 ± 0.49	0.010 (−0.064, 0.083)	2.44 (2.38, 2.51)				CSR vs. CRS <0.001
	CSR	2.37 ± 0.34	2.08 ± 0.43	−0.287 (−0.356, −0.218)	2.14 (2.08, 2.21)				CON vs. CRS = 1.000
BMI	CON	27.06 ± 0.93	27.32 ± 1.04	0.27 (−0.13, 0.66)	27.45 (27.13, 27.77)	34.08	<0.001	0.553	CRS vs. CON <0.001
	CRS	28.03 ± 3.14	26.45 ± 3.09	−1.60 (−1.88, −1.32)	25.60 (25.27, 25.93)				CSR vs. CON <0.001
	CSR	26.50 ± 2.50	25.54 ± 2.69	−0.97 (−1.28, −0.66)	26.21 (25.88, 26.53)				CRS vs. CSR = 0.039
BF%	CON	40.83 ± 10.65	41.39 ± 10.32	0.56 (−0.21, 1.33)	40.84 (39.60, 42.08)	30.24	<0.001	0.524	CRS vs. CON <0.001
	CRS	41.05 ± 3.71	35.23 ± 4.97	−5.63 (−7.55, −3.71)	34.47 (33.20, 35.74)				CSR vs. CON <0.001
	CSR	38.98 ± 5.90	34.22 ± 7.03	−4.76 (−5.85, −3.67)	35.50 (34.25, 36.74)				CRS vs. CSR = 0.757
Weight	CON	70.19 ± 7.22	71.83 ± 6.69	1.67 (0.76, 2.59)	71.27 (70.52, 72.03)	36.36	<0.001	0.569	CSR vs. CON <0.001
	CRS	74.03 ± 13.18	71.32 ± 13.19	2.71 (1.92, 3.50)	72.33 (71.55, 73.11)				CSR vs. CRS <0.001
	CSR	67.37 ± 10.51	65.68 ± 10.85	−1.68 (−2.33, −1.04)	67.88 (67.12, 68.64)				CON vs. CRS = 0.170
WC	CON	91.10 ± 7.26	92.10 ± 7.09	0.97 (0.36, 1.59)	92.29 (91.43, 93.15)	32.80	<0.001	0.544	CRS vs. CON <0.001
	CRS	94.05 ± 8.60	90.47 ± 9.05	−3.54 (−4.54, −2.54)	87.86 (86.96, 88.76)				CSR vs. CON <0.001
	CSR	89.05 ± 5.91	86.00 ± 5.53	−3.05 (−4.09, −2.01)	88.20 (87.33, 89.08)				CRS vs. CSR = 1.000
WHR	CON	0.923 ± 0.083	0.956 ± 0.083	0.004 (−0.014, 0.022)	0.936 (0.924, 0.948)	11.34	<0.001	0.292	CRS vs. CON = 0.012
	CRS	0.957 ± 0.055	0.930 ± 0.055	−0.027 (−0.038, −0.017)	0.909 (0.897, 0.922)				CSR vs. CON <0.001
	CSR	0.922 ± 0.044	0.885 ± 0.043	−0.036 (−0.045, −0.027)	0.896 (0.884, 0.908)				CRS vs. CSR = 0.411
FFM	CON	41,404.1 ± 7,855.3	42062.5 ± 7969.6	658.5 (320.8, 996.1)	42,606.6 (41,910.8, 43,302.5)	6.77	0.002	0.198	CRS vs. CON = 0.003
	CRS	43,485.4 ± 7,309.6	45,861.7 ± 7,044.5	2,376.3 (1,227.1, 3,525.6)	44,357.2 (43,638.9, 45,075.5)				CSR vs. CON = 0.027
	CSR	41,057.6 ± 7,159.1	43,047.2 ± 7,409.8	1,989.6 (1,498.8, 2,480.4)	43,932.3 (43,235.3, 44,629.3)				CRS vs. CSR = 1.000

**Bone health: BMD, BMC, *Z* score**									

BMD	CON	0.973 ± 0.067	1.045 ± 0.072	0.072 (0.055, 0.089)	1.044 (1.029, 1.059)	5.35	0.008	0.163	CSR vs. CON = 0.007
	CRS	0.984 ± 0.097	1.031 ± 0.085	0.047 (0.029, 0.065)	1.021 (1.005, 1.036)				CON vs. CRS = 0.091
	CSR	0.960 ± 0.093	1.000 ± 0.090	0.040 (0.026, 0.054)	1.011 (0.996, 1.026)				CRS vs. CSR = 1.000
BMC	CON	1,929.9 ± 306.4	2,036.4 ± 287.0	106.5 (61.7, 151.4)	2,039.3 (2,002.3, 2076.2)	0.18	0.835	0.007	No significant pairwise differences
	CRS	1,958.7 ± 323.9	2,069.9 ± 307.7	111.2 (72.7, 149.7)	2,045.4 (2,007.4, 2,083.4)				
	CSR	1,911.4 ± 267.8	2,034.4 ± 296.0	123.0 (89.6, 156.4)	2,054.8 (2,017.8, 2,091.9)				
*Z* score	CON	2.17 ± 0.76	2.47 ± 0.69	0.30 (0.20, 0.41)	2.48 (2.38, 2.58)	0.09	0.917	0.003	No significant pairwise differences
	CRS	2.10 ± 0.74	2.44 ± 0.64	0.34 (0.21, 0.47)	2.51 (2.41, 2.61)				
	CSR	2.27 ± 0.79	2.57 ± 0.79	0.30 (0.20, 0.41)	2.49 (2.39, 2.59)				

**Secondary fitness and functional outcomes: 20-m SRT, standing long jump, rope skipping, sit-ups, grip strength, vital capacity**									

20-m SRT	CON	23.55 ± 7.29	27.00 ± 7.99	3.45 (2.38, 4.52)	26.75 (24.66, 28.84)	24.74	<0.001	0.474	CRS vs. CON <0.001
	CRS	22.53 ± 8.22	36.11 ± 11.68	13.58 (10.46, 16.70)	36.98 (34.84, 39.13)				CSR vs. CON <0.001
	CSR	23.85 ± 9.78	34.40 ± 11.41	10.55 (8.43, 12.67)	33.82 (31.72, 35.91)				CRS vs. CSR = 0.117
Standing long jump	CON	144.62 ± 25.62	149.54 ± 23.20	4.92 (2.25, 7.59)	149.19 (144.02, 154.36)	9.05	<0.001	0.248	CSR vs. CON <0.001
	CRS	145.63 ± 22.16	157.53 ± 22.87	11.89 (6.97, 16.82)	156.27 (150.96, 161.57)				CRS vs. CON = 0.182
	CSR	142.50 ± 19.59	163.15 ± 23.72	20.65 (12.97, 28.33)	164.70 (159.53, 169.87)				CRS vs. CSR = 0.080
Rope skipping	CON	135.10 ± 31.71	142.25 ± 25.49	7.15 (−0.95, 15.25)	142.89 (135.96, 149.82)	5.94	0.005	0.178	CRS vs. CON = 0.025
	CRS	139.32 ± 28.25	158.58 ± 25.48	19.26 (9.03, 29.50)	156.43 (149.31, 163.55)				CSR vs. CON = 0.007
	CSR	133.95 ± 29.01	157.00 ± 23.82	23.05 (15.48, 30.62)	158.40 (151.47, 165.33)				CRS vs. CSR = 1.000
Sit-ups	CON	28.32 ± 6.92	29.59 ± 6.71	1.27 (−1.29, 3.83)	29.65 (27.32, 31.99)	6.43	0.003	0.189	CRS vs. CON = 0.043
	CRS	28.05 ± 8.31	33.63 ± 9.47	5.58 (2.51, 8.65)	33.88 (31.49, 36.28)				CSR vs. CON = 0.003
	CSR	28.85 ± 7.64	35.65 ± 5.52	6.80 (4.38, 9.22)	35.35 (33.01, 37.68)				CRS vs. CSR = 1.000
Grip strength	CON	23.91 ± 5.08	26.78 ± 5.40	2.87 (2.04, 3.70)	26.76 (25.48, 28.03)	0.18	0.839	0.006	No significant pairwise differences
	CRS	24.63 ± 5.43	27.98 ± 5.58	3.35 (2.03, 4.67)	27.29 (25.98, 28.60)				
	CSR	23.16 ± 5.67	26.26 ± 6.22	3.10 (1.38, 4.83)	26.94 (25.66, 28.22)				
Vital capacity	CON	3,139.25 ± 646.86	3,299.35 ± 622.20	160.10 (−52.88, 373.08)	3,248.41 (3,102.07, 3,394.75)	2.92	0.063	0.096	No significant pairwise differences
	CRS	3,057.21 ± 620.72	3,463.32 ± 564.45	406.11 (273.38, 538.83)	3,478.22 (3,328.33, 3,628.11)				
	CSR	3,029.95 ± 599.81	3,414.20 ± 595.83	384.25 (257.64, 510.86)	3,450.98 (3,304.77, 3,597.20)				

**Cardiovascular and physiological outcomes: Resting heart rate, SBP, DBP, hip circumference**									

Resting heart rate	CON	74.25 ± 9.39	72.60 ± 9.67	−1.65 (−3.77, 0.47)	74.42 (71.37, 77.47)	6.74	0.002	0.197	CRS vs. CON = 0.002
	CRS	79.56 ± 9.18	68.39 ± 8.31	−11.17 (−15.61, −6.72)	66.57 (63.44, 69.70)				CSR vs. CON = 0.036
	CSR	77.04 ± 10.59	68.95 ± 10.19	−8.09 (−11.65, −4.54)	68.86 (65.84, 71.87)				CRS vs. CSR = 0.890
SBP	CON	117.05 ± 6.89	115.70 ± 11.02	−1.35 (−5.36, 2.66)	115.85 (111.19, 120.50)	0.69	0.508	0.024	No significant pairwise differences
	CRS	117.95 ± 8.09	117.79 ± 13.75	−0.16 (−6.30, 5.98)	117.34 (112.56, 122.12)				
	CSR	116.85 ± 11.05	113.20 ± 10.66	−3.65 (−8.58, 1.28)	113.48 (108.83, 118.13)				
DBP	CON	67.15 ± 6.68	67.25 ± 7.22	0.10 (−3.09, 3.29)	67.72 (64.19, 71.24)	1.12	0.333	0.039	No significant pairwise differences
	CRS	68.26 ± 6.24	68.58 ± 8.34	0.32 (−3.81, 4.44)	68.65 (65.05, 72.24)				
	CSR	69.95 ± 7.33	65.55 ± 8.73	−4.40 (−9.47, 0.67)	65.02 (61.48, 68.55)				
Hip circumference	CON	99.05 ± 8.37	99.62 ± 7.59	0.57 (−1.06, 2.20)	98.71 (97.71, 99.70)	3.01	0.057	0.099	No significant pairwise differences
	CRS	98.32 ± 8.92	97.32 ± 8.75	−1.00 (−1.60, −0.40)	97.06 (96.04, 98.08)				
	CSR	96.74 ± 6.40	97.23 ± 5.78	0.49 (−0.26, 1.24)	98.39 (97.39, 99.38)				

Results for WHR, FFM, vital capacity, standing long jump, 20-m SRT, VO_2_max, hip circumference, and resting heart rate should be interpreted with caution because the homogeneity of variance assumption was violated for these outcomes.

Attendance was high in both exercise groups, averaging 35.79 ± 0.71 of 36 sessions (99.42 ± 1.98%) in CRS and 35.90 ± 0.31 of 36 sessions (99.72 ± 0.85%) in CSR, with no significant between-group difference (*p* = 0.709).

### Primary outcome: VO_2_max

After adjusting for baseline VO_2_max, a significant group effect was observed for post-intervention VO_2_max, F(2, 55) = 45.881, *p* < 0.001, partial η^2^ = 0.625. The adjusted post-intervention VO_2_max values were 40.557 mL kg^−1^·min^−1^ (95% confidence interval [CI], 39.921 to 41.193) for CON, 44.836 mL kg^−1^·min^−1^ (95% CI, 44.181 to 45.491) for CRS, and 43.631 mL kg^−1^·min^−1^ (95% CI, 43.006 to 44.256) for CSR. Bonferroni-adjusted pairwise comparisons showed that both CRS and CSR had significantly higher VO_2_max than CON (CRS vs. CON, mean difference = 4.279, 95% CI, 3.132 to 5.426, *p* < 0.001; CSR vs. CON, mean difference = 3.074, 95% CI, 1.977 to 4.171, *p* < 0.001). In addition, CRS showed significantly higher VO_2_max than CSR (mean difference = 1.205, 95% CI, 0.088 to 2.322, *p* = 0.030).

Descriptively, VO_2_max increased in all three groups, with mean changes of 0.95 (95% CI, 0.52 to 1.38) in CON, 5.28 (95% CI, 4.49 to 6.07) in CRS, and 4.05 (95% CI, 3.35 to 4.75) in CSR. Pairwise effect sizes based on change scores indicated large between-group effects for both training groups compared with CON (Hedges’ g = 3.206 for CRS vs. CON; 2.442 for CSR vs. CON), whereas the difference between CRS and CSR was smaller (g = 0.772).

As a sensitivity analysis, ANCOVA was repeated with VO_2_max measurement method (direct measurement vs. estimated from the 20-m shuttle run test) included as an additional fixed factor. The group effect remained significant after adjustment for baseline VO_2_max and measurement method, F(2, 52) = 43.840, *p* < 0.001, partial η^2^ = 0.628. Neither the main effect of measurement method, F(1, 52) = 1.216, *p* = 0.275, nor the group × method interaction, F(2, 52) = 0.500, *p* = 0.609, was significant, indicating that the between-group differences in VO_2_max were robust and were not materially influenced by measurement method.

The distribution of VO_2_max measurement methods was similar across groups (CON, 10 direct/10 estimated; CRS, 10 direct/9 estimated; CSR, 10 direct/10 estimated; χ^2^ = 0.036, *p* = 0.982). At baseline, VO_2_max did not significantly differ between participants assessed by direct measurement and those assessed by estimation (40.52 ± 4.04 vs. 38.62 ± 3.33 mL kg^−1^·min^−1^, *p* = 0.054), although values were numerically higher in the direct-measurement subgroup.

### Body composition outcomes

After adjustment for baseline values, significant group effects were observed for BMI-z, BMI, body fat percentage (BF%), body weight, waist circumference (WC), waist-to-hip ratio (WHR), and fat-free mass (FFM).

For BMI-z, a significant group effect was found, F(2, 55) = 23.993, *p* < 0.001, partial η^2^ = 0.466. The adjusted post-intervention BMI-z values were 2.401 (95% CI, 2.336 to 2.467) in CON, 2.442 (95% CI, 2.375 to 2.509) in CRS, and 2.144 (95% CI, 2.078 to 2.209) in CSR. Bonferroni-adjusted pairwise comparisons showed that CSR had significantly lower BMI-z than both CON and CRS (both *p* < 0.001), whereas no significant difference was observed between CON and CRS (*p* = 1.000).

For BMI, the group effect was also significant, F(2, 55) = 34.084, *p* < 0.001, partial η^2^ = 0.553. The adjusted post-intervention BMI values were 27.452 kg/m^2^ (95% CI, 27.133 to 27.771) in CON, 25.601 kg/m^2^ (95% CI, 25.267 to 25.935) in CRS, and 26.207 kg/m^2^ (95% CI, 25.883 to 26.530) in CSR. Pairwise comparisons showed that both training groups had significantly lower BMI than CON (both *p* < 0.001), and CRS also had significantly lower BMI than CSR (*p* = 0.039).

A significant group effect was observed for BF%, F(2, 55) = 30.243, *p* < 0.001, partial η^2^ = 0.524. Adjusted post-intervention BF% was 40.840% (95% CI, 39.601 to 42.079) in CON, 34.467% (95% CI, 33.195 to 35.739) in CRS, and 35.497% (95% CI, 34.253 to 36.741) in CSR. Both CRS and CSR had significantly lower BF% than CON (both *p* < 0.001), whereas the difference between CRS and CSR was not significant (*p* = 0.757).

Similarly, body weight differed significantly among groups after adjustment, F(2, 55) = 36.355, *p* < 0.001, partial η^2^ = 0.569. The adjusted post-intervention body weights were 71.274 kg (95% CI, 70.517 to 72.031) in CON, 72.329 kg (95% CI, 71.549 to 73.109) in CRS, and 67.881 kg (95% CI, 67.119 to 68.644) in CSR. CSR had significantly lower body weight than both CON and CRS (both *p* < 0.001), whereas CON and CRS did not differ significantly (*p* = 0.170).

For central adiposity, a significant group effect was found for WC, F(2, 55) = 32.799, *p* < 0.001, partial η^2^ = 0.544. Adjusted post-intervention WC was 92.289 cm (95% CI, 91.432 to 93.146) in CON, 87.860 cm (95% CI, 86.961 to 88.759) in CRS, and 88.204 cm (95% CI, 87.333 to 89.075) in CSR. Both training groups had significantly lower WC than CON (both *p* < 0.001), with no significant difference between CRS and CSR (*p* = 1.000). For WHR, the group effect was also significant, F(2, 55) = 11.339, *p* < 0.001, partial η^2^ = 0.292. Adjusted post-intervention WHR values were 0.936 (95% CI, 0.924 to 0.948) in CON, 0.909 (95% CI, 0.897 to 0.922) in CRS, and 0.896 (95% CI, 0.884 to 0.908) in CSR. CON showed significantly higher WHR than both CRS (*p* = 0.012) and CSR (*p* < 0.001), whereas CRS and CSR did not differ significantly (*p* = 0.411).

A significant group effect was additionally observed for FFM, F(2, 55) = 6.771, *p* = 0.002, partial η^2^ = 0.198. Adjusted post-intervention FFM was 42,606.624 g (95% CI, 41,910.788 to 43,302.461) in CON, 44,357.234 g (95% CI, 43,638.946 to 45,075.522) in CRS, and 43,932.303 g (95% CI, 43,235.342 to 44,629.263) in CSR. Both CRS and CSR had significantly higher FFM than CON (*p* = 0.003 and *p* = 0.027, respectively), while CRS and CSR did not differ significantly (*p* = 1.000).

Descriptively, changes in adiposity-related indicators generally favored the two training groups over CON. For example, mean changes in BMI were 0.27 (95% CI, −0.13 to 0.66) in CON, −1.60 (95% CI, −1.88 to −1.32) in CRS, and −0.97 (95% CI, −1.28 to −0.66) in CSR, whereas mean changes in BF% were 0.56 (95% CI, −0.21 to 1.33), −5.63 (95% CI, −7.55 to −3.71), and −4.76 (95% CI, −5.85 to −3.67) in CON, CRS, and CSR, respectively. Pairwise effect sizes based on change scores were large for several body composition outcomes, particularly BMI, BF%, and WC, when either training group was compared with CON.

### Bone health outcomes

After adjustment for baseline values, a significant group effect was observed for BMD, whereas no significant group effects were found for BMC or *Z* score.

For BMD, the ANCOVA showed a significant group effect, F(2, 55) = 5.350, *p* = 0.008, partial η^2^ = 0.163. The adjusted post-intervention BMD values were 1.044 g/cm^2^ (95% CI, 1.029 to 1.059) in CON, 1.021 g/cm^2^ (95% CI, 1.005 to 1.036) in CRS, and 1.011 g/cm^2^ (95% CI, 0.996 to 1.026) in CSR. Bonferroni-adjusted pairwise comparisons indicated that CON had significantly higher BMD than CSR (*p* = 0.007), whereas no significant differences were found between CON and CRS (*p* = 0.091) or between CRS and CSR (*p* = 1.000).

By contrast, no significant group effect was found for BMC, F(2, 55) = 0.181, *p* = 0.835, partial η^2^ = 0.007. The adjusted post-intervention BMC values were 2,039.261 g (95% CI, 2,002.283 to 2,076.240) in CON, 2,045.369 g (95% CI, 2,007.382 to 2,083.355) in CRS, and 2,054.842 g (95% CI, 2,017.830 to 2,091.854) in CSR, with no significant pairwise differences.

Similarly, no significant group effect was observed for *Z* score, F(2, 55) = 0.086, *p* = 0.917, partial η^2^ = 0.003. The adjusted post-intervention Z-scores were 2.481 (95% CI, 2.381 to 2.582) in CON, 2.511 (95% CI, 2.408 to 2.614) in CRS, and 2.493 (95% CI, 2.393 to 2.593) in CSR, and no significant pairwise differences were detected.

Descriptively, all three groups showed positive mean changes in bone-related outcomes over the intervention period. Mean changes in BMD were 0.072 (95% CI, 0.055 to 0.089) in CON, 0.047 (95% CI, 0.029 to 0.065) in CRS, and 0.040 (95% CI, 0.026 to 0.054) in CSR. Corresponding mean changes in BMC were 106.5 g (95% CI, 61.7 to 151.4) in CON, 111.2 g (95% CI, 72.7 to 149.7) in CRS, and 123.0 g (95% CI, 89.6 to 156.4) in CSR. Mean changes in *Z* score were 0.305 (95% CI, 0.196 to 0.414) in CON, 0.342 (95% CI, 0.213 to 0.471) in CRS, and 0.305 (95% CI, 0.205 to 0.405) in CSR.

### Secondary fitness and functional outcomes

After adjustment for baseline values, significant group effects were observed for 20-m shuttle run test (20-m SRT), standing long jump, rope skipping, and sit-ups, whereas no significant group effects were found for grip strength or vital capacity.

For 20-m SRT, ANCOVA showed a significant group effect, F(2, 55) = 24.739, *p* < 0.001, partial η^2^ = 0.474. The adjusted post-intervention 20-m SRT values were 26.748 laps (95% CI, 24.657 to 28.839) in CON, 36.985 laps (95% CI, 34.837 to 39.133) in CRS, and 33.816 laps (95% CI, 31.724 to 35.908) in CSR. Bonferroni-adjusted pairwise comparisons showed that both CRS and CSR performed significantly better than CON (both *p* < 0.001), whereas the difference between CRS and CSR was not significant (*p* = 0.117).

For standing long jump, a significant group effect was also detected, F(2, 55) = 9.054, *p* < 0.001, partial η^2^ = 0.248. The adjusted post-intervention values were 149.188 cm (95% CI, 144.020 to 154.355) in CON, 156.266 cm (95% CI, 150.961 to 161.571) in CRS, and 164.699 cm (95% CI, 159.527 to 169.872) in CSR. Pairwise comparisons indicated that CSR performed significantly better than CON (*p* < 0.001), whereas the differences between CRS and CON (*p* = 0.182) and between CRS and CSR (*p* = 0.080) were not statistically significant.

A significant group effect was further observed for rope skipping, F(2, 55) = 5.939, *p* = 0.005, partial η^2^ = 0.178. The adjusted post-intervention rope-skipping values were 142.891 counts/min (95% CI, 135.965 to 149.817) in CON, 156.429 counts/min (95% CI, 149.310 to 163.547) in CRS, and 158.402 counts/min (95% CI, 151.471 to 165.333) in CSR. Both CRS and CSR showed significantly higher rope-skipping performance than CON (*p* = 0.025 and *p* = 0.007, respectively), with no significant difference between CRS and CSR (*p* = 1.000).

Similarly, sit-ups differed significantly among groups, F(2, 55) = 6.426, *p* = 0.003, partial η^2^ = 0.189. The adjusted post-intervention sit-up values were 29.654 counts/min (95% CI, 27.320 to 31.988) in CON, 33.880 counts/min (95% CI, 31.485 to 36.276) in CRS, and 35.348 counts/min (95% CI, 33.013 to 37.684) in CSR. Both CRS and CSR performed significantly better than CON (*p* = 0.043 and *p* = 0.003, respectively), whereas no significant difference was observed between CRS and CSR (*p* = 1.000).

By contrast, no significant group effect was found for grip strength, F(2, 55) = 0.176, *p* = 0.839, partial η^2^ = 0.006. The adjusted post-intervention grip strength values were 26.758 kg (95% CI, 25.484 to 28.032) in CON, 27.291 kg (95% CI, 25.980 to 28.602) in CRS, and 26.938 kg (95% CI, 25.660 to 28.216) in CSR, with no significant pairwise differences. Likewise, no significant group effect was observed for vital capacity, F(2, 55) = 2.917, *p* = 0.063, partial η^2^ = 0.096. The adjusted post-intervention vital capacity values were 3,248.409 mL (95% CI, 3,102.067 to 3,394.751) in CON, 3,478.219 mL (95% CI, 3,328.331 to 3,628.108) in CRS, and 3,450.983 mL (95% CI, 3,304.770 to 3,597.195) in CSR, and no significant pairwise differences were found.

Descriptively, the two training groups generally demonstrated greater improvements than CON in several functional fitness outcomes. Mean changes in 20-m SRT were 3.45 (95% CI, 2.38 to 4.52) in CON, 13.58 (95% CI, 10.46 to 16.70) in CRS, and 10.55 (95% CI, 8.43 to 12.67) in CSR. Mean changes in standing long jump were 4.92 cm (95% CI, 2.25 to 7.59) in CON, 11.89 cm (95% CI, 6.97 to 16.82) in CRS, and 20.65 cm (95% CI, 12.97 to 28.33) in CSR. Mean changes in rope skipping were 7.15 counts/min (95% CI, −0.95 to 15.25) in CON, 19.26 counts/min (95% CI, 9.03 to 29.50) in CRS, and 23.05 counts/min (95% CI, 15.48 to 30.62) in CSR, whereas mean changes in sit-ups were 1.27 counts/min (95% CI, −1.29 to 3.83), 5.58 counts/min (95% CI, 2.51 to 8.65), and 6.80 counts/min (95% CI, 4.38 to 9.22) in CON, CRS, and CSR, respectively.

### Cardiovascular and physiological outcomes

After adjustment for baseline values, a significant group effect was observed for resting heart rate, whereas no significant group effects were found for systolic blood pressure (SBP), diastolic blood pressure (DBP), or hip circumference.

For resting heart rate, ANCOVA showed a significant group effect, F(2, 55) = 6.743, *p* = 0.002, partial η^2^ = 0.197. The adjusted post-intervention resting heart rates were 74.421 bpm (95% CI, 71.367 to 77.474) in CON, 66.573 bpm (95% CI, 63.442 to 69.703) in CRS, and 68.857 bpm (95% CI, 65.843 to 71.871) in CSR. Bonferroni-adjusted pairwise comparisons indicated that both CRS and CSR had significantly lower resting heart rate than CON (CRS vs. CON, *p* = 0.002; CSR vs. CON, *p* = 0.036), whereas the difference between CRS and CSR was not significant (*p* = 0.890).

No significant group effect was found for SBP, F(2, 55) = 0.687, *p* = 0.508, partial η^2^ = 0.024. The adjusted post-intervention SBP values were 115.847 mmHg (95% CI, 111.194 to 120.499) in CON, 117.340 mmHg (95% CI, 112.563 to 122.118) in CRS, and 113.480 mmHg (95% CI, 108.826 to 118.134) in CSR, with no significant pairwise differences. Similarly, DBP did not differ significantly among groups, F(2, 55) = 1.122, *p* = 0.333, partial η^2^ = 0.039. The adjusted post-intervention DBP values were 67.717 mmHg (95% CI, 64.189 to 71.244) in CON, 68.648 mmHg (95% CI, 65.053 to 72.244) in CRS, and 65.018 mmHg (95% CI, 61.483 to 68.552) in CSR, with no significant pairwise differences.

For hip circumference, the group effect did not reach statistical significance, F(2, 55) = 3.013, *p* = 0.057, partial η^2^ = 0.099. The adjusted post-intervention hip circumference values were 98.707 cm (95% CI, 97.713 to 99.701) in CON, 97.060 cm (95% CI, 96.043 to 98.078) in CRS, and 98.385 cm (95% CI, 97.390 to 99.381) in CSR, with no significant pairwise differences.

Descriptively, mean changes in resting heart rate were −1.65 bpm (95% CI, −3.77 to 0.47) in CON, −11.17 bpm (95% CI, −15.61 to −6.72) in CRS, and −8.09 bpm (95% CI, −11.65 to −4.54) in CSR. Mean changes in SBP were −1.35 mmHg (95% CI, −5.36 to 2.66) in CON, −0.16 mmHg (95% CI, −6.30 to 5.98) in CRS, and −3.65 mmHg (95% CI, −8.58 to 1.28) in CSR. Mean changes in DBP were 0.10 mmHg (95% CI, −3.09 to 3.29) in CON, 0.32 mmHg (95% CI, −3.81 to 4.44) in CRS, and −4.40 mmHg (95% CI, −9.47 to 0.67) in CSR. Mean changes in hip circumference were 0.57 cm (95% CI, −1.06 to 2.20) in CON, −1.00 cm (95% CI, −1.60 to −0.40) in CRS, and 0.49 cm (95% CI, −0.26 to 1.24) in CSR.

## Discussion

### Principal findings

The present study compared two within-session exercise orders in a school-based program combining repeated sprint exercise and bodyweight resistance exercise in adolescents with obesity. The main finding was that both training sequences produced more favorable changes than the non-exercising control condition in several health-related outcomes, particularly cardiorespiratory fitness and body composition. Overall, the order effect appeared to be limited, as most outcomes showed a similar pattern in the two training groups. However, exercise order was not entirely neutral. A modest but meaningful between-sequence difference was observed for VO_2_max, with the CRS group showing a greater improvement than the CSR group.

For body composition, both training groups generally performed better than CON, especially for BMI, BF%, WC, and related adiposity indicators. These findings suggest that, under matched training duration and school-based delivery conditions, the inclusion of both sprint-based and bodyweight resistance components can provide clear benefits for adolescents with obesity, regardless of the specific order in which the two components are arranged. At the same time, the differences between CRS and CSR for most body composition outcomes were small or inconsistent, indicating that exercise order may play a secondary role compared with the overall training stimulus itself.

For bone-related outcomes, the pattern was less clear. BMD showed a significant group effect, whereas BMC and *Z* score did not. Taken together, these findings suggest that the intervention may have had some influence on skeletal adaptation, but the evidence for a robust order-specific effect on bone health was limited. This point should be interpreted cautiously, particularly given the age of the participants and the likelihood that normal pubertal growth contributed to changes in bone outcomes across all groups.

From a practical perspective, the present findings support the use of time-efficient, school-based high-intensity combined training for adolescents with obesity.^9^^,^^10^^,^^11^ The two exercise sequences were broadly comparable across most outcomes, which is encouraging for real-world physical education settings where class organization, space, and equipment constraints often require flexible programming.^11^^,^^18^ However, if improving cardiorespiratory fitness is the primary objective, the present results suggest that performing the resistance component before the sprint component may offer a small advantage.

Because the participants were undergoing normal pubertal growth, some increases in the control group were expected and should not be interpreted as evidence of an intervention-like effect.^4^^,^^6^^,^^22^ For this reason, the results are best interpreted on the basis of adjusted between-group differences rather than within-group changes alone. In practical terms, the observed advantages for VO_2_max, BMI/BF%, and WC are particularly relevant, as they indicate improvements in aerobic fitness, adiposity, and central obesity risk.^2^^,^^3^^,^^7^ These changes support the potential clinical and school-based relevance of the intervention for adolescents with obesity.^2^^,^^11^

### Primary outcome: VO_2_max

VO_2_max was selected as the primary outcome because cardiorespiratory fitness is one of the most clinically relevant health indicators in youth with obesity.^2^^,^^7^^,^^8^ In the present study, both training groups showed clear advantages over the control group, and the CRS group also demonstrated a significantly greater improvement than the CSR group. This pattern suggests that while both exercise sequences were effective for improving aerobic fitness, the order of the two training components may still matter when VO_2_max is considered the main target.

One possible explanation is that performing the bodyweight resistance component before the sprint component may have allowed participants to maintain a more effective physiological stimulus across the full session.^19^^,^^20^ Although both sequences contained the same exercise components and overall duration, the order in which fatigue accumulated may have differed. In the CSR sequence, participants completed the repeated sprint task first, which may have increased acute fatigue before the subsequent resistance-based component. In contrast, the CRS sequence may have distributed effort differently across the lesson, allowing the sprint portion to be completed with a more favorable pacing pattern or more consistent movement quality. Because VO_2_max adaptation is highly sensitive to the quality and intensity of aerobic work, even a modest difference in within-session fatigue distribution could contribute to the group difference observed here.^20^^,^^23^

At the same time, the between-sequence difference in VO_2_max should not be overstated. Both CRS and CSR improved substantially compared with CON, indicating that the presence of a combined high-intensity training stimulus was more important than sequence alone.^19^^,^^21^^,^^24^ In other words, the overall intervention effect was strong, whereas the order effect was more selective. This interpretation is consistent with the broader pattern of findings in the present study, where most outcomes favored both training groups over the control group, but only a limited number of outcomes differentiated CRS from CSR.

An additional concern raised during peer review was whether the VO_2_max findings might have been influenced by the use of two assessment approaches, namely direct measurement and values estimated from the 20-m shuttle run test. To address this issue, a sensitivity analysis was performed with measurement method included as an additional fixed factor. The group effect remained significant, whereas neither the main effect of measurement method nor the group × method interaction was significant. This provides some reassurance that the observed difference in VO_2_max was not simply an artifact of measurement-method bias.

From a practical standpoint, this finding has direct relevance for school-based exercise programming. If the primary goal of a physical education intervention is to improve cardiorespiratory fitness in adolescents with obesity, arranging the lesson so that the bodyweight resistance component precedes the sprint component may be preferable.^18^^,^^19^ However, because the two training groups were broadly similar across many other outcomes, the choice of sequence can still remain flexible when lesson management, class organization, or space limitations make one order easier to implement than the other.

### Body composition adaptations

In addition to the improvement in cardiorespiratory fitness, both training sequences were associated with favorable changes in several body composition outcomes. Compared with the control group, the two training groups generally showed lower post-intervention values for BMI, BF%, WC, and related adiposity indicators after adjustment for baseline levels. This overall pattern suggests that combining repeated sprint exercise with bodyweight resistance exercise can produce meaningful benefits for adiposity management in adolescents with obesity under real-world school conditions.^7^^,^^11^^,^^21^^,^^24^

An important observation was that the differences between CRS and CSR were much smaller for body composition than for VO_2_max. For most adiposity-related outcomes, both intervention groups outperformed CON, whereas the contrast between the two exercise orders was either modest or not statistically significant. This suggests that, for body composition, the total training stimulus may be more influential than the exact sequence in which the sprint and resistance components are performed.^19^^,^^21^ In practical terms, as long as both components are included and delivered consistently, the overall metabolic demand of the session may be sufficient to induce favorable changes in fat-related outcomes.

The reductions observed in BMI, BF%, and WC are also noteworthy because these indicators reflect slightly different aspects of obesity-related risk. BMI and BMI-z capture general adiposity status, whereas BF% and WC are more closely related to body fat accumulation and central adiposity.^2^^,^^3^ The fact that improvements were seen across several of these indicators strengthens the interpretation that the intervention was not only effective at increasing energy expenditure during class, but also contributed to broader changes in body composition over the 12-week period.

The findings for FFM should be interpreted somewhat differently. Both training groups showed higher adjusted post-intervention FFM than CON, whereas the difference between CRS and CSR was not significant. This pattern suggests that the combined training format may have supported the maintenance or modest development of lean tissue while body fat was reduced. In a pediatric obesity context, this is an important point, because successful intervention is not simply about lowering body mass, but about improving body composition quality. A reduction in adiposity accompanied by preservation or improvement of FFM is generally more desirable than weight loss alone.^8^^,^^25^

At the same time, the body composition results do not support a strong order-dependent effect. Although CRS showed slightly more favorable changes than CSR for some indicators, these differences were not consistent across outcomes. Taken together, the results suggest that exercise order may have some influence on selected endpoints, but it is unlikely to be the main determinant of body composition adaptation in this context. For school-based obesity interventions, this may actually be encouraging, as it implies that teachers can retain a degree of flexibility in lesson sequencing without substantially compromising body composition benefits.^11^^,^^18^

### Bone health outcomes

The bone-related findings were more mixed and should be interpreted with caution.^22^^,^^26^ Among the three bone outcomes examined, only BMD showed a significant group effect, whereas BMC and *Z* score did not differ significantly among groups. This pattern suggests that the intervention may have had some influence on skeletal adaptation, but the overall evidence for a clear and consistent order-related effect on bone health was limited.

One possible explanation is that the osteogenic stimulus provided by the intervention was sufficient to support some skeletal adaptation, but not strong or specific enough to produce consistent between-group differences across all bone indicators. Both repeated sprint exercise and bodyweight resistance exercise involve weight-bearing and impact-loading components, which may be beneficial for bone development during adolescence.^4^^,^^5^^,^^27^ However, the skeletal response to exercise is influenced by multiple factors, including loading magnitude, loading rate, maturation status, nutritional status, and habitual physical activity outside the intervention.^4^^,^^22^^,^^27^ Under these conditions, it is plausible that the intervention contributed to bone adaptation, but that the effect was modest and not highly sensitive to within-session exercise order.

Another issue is that all groups, including the control group, showed positive changes in bone-related variables over time. This is not surprising given that the participants were adolescents, a period characterized by rapid growth and active skeletal development.^6^^,^^22^ Therefore, at least part of the observed improvement in BMD, BMC, and *Z* score may reflect normal growth rather than a pure training effect.^4^^,^^22^^,^^26^ This is particularly relevant when interpreting the significant result for BMD, because the absence of corresponding differences in BMC and *Z* score makes it difficult to argue for a strong, isolated intervention-specific bone benefit.

A further limitation is that maturation-related variables were not assessed in the present study. Indicators such as Tanner stage, maturity offset, or bone age were not available, and therefore, additional adjustment for pubertal growth was not feasible. As a result, residual confounding by growth and maturation cannot be ruled out. This should be acknowledged when interpreting the bone findings, especially in a sample of adolescents with obesity undergoing normal developmental change.

From a practical perspective, the bone results should be viewed as preliminary rather than definitive. The present intervention does not appear to have negatively affected skeletal development, and the positive direction of change across groups is reassuring. However, the evidence is not strong enough to conclude that one training order offers a clear advantage for bone health. Longer intervention periods, direct assessment of maturation, and more detailed monitoring of loading exposure would be needed to clarify whether exercise sequence meaningfully influences skeletal outcomes in this population.^22^^,^^26^^,^^28^

### Secondary fitness and physiological outcomes

Beyond VO_2_max and body composition, the intervention also influenced several secondary fitness and physiological outcomes. In general, the two training groups showed more favorable patterns than the control group in functional fitness tests, whereas the effects on blood pressure-related variables were limited. This overall pattern suggests that the school-based combined training program was able to improve multiple aspects of physical function, but that not all physiological outcomes responded to the same extent over the 12-week period.^9^^,^^11^^,^^13^

Among the secondary fitness outcomes, both intervention groups performed better than the control group in 20-m SRT, rope skipping, and sit-ups after adjustment for baseline values. These findings are broadly consistent with the nature of the intervention itself.^10^^,^^11^ Both repeated sprint exercise and bodyweight resistance exercise require repeated effort, lower-limb power production, movement coordination, and local muscular endurance.^13^^,^^23^ It is therefore reasonable that the training stimulus transferred to performance in shuttle running, repeated jumping or skipping, and trunk endurance tasks. The fact that the two intervention groups were generally similar to each other across these outcomes again supports the view that the combined training stimulus, rather than exercise order alone, was the dominant factor.

The standing long jump findings showed a slightly different pattern. Although the overall group effect was significant, the clearest difference was between CSR and CON, while the contrast between CRS and CSR did not reach significance. This may indicate that lower-limb explosive performance was somewhat more variable across participants and may have been influenced by factors such as baseline motor competence, motivation during testing, or day-to-day fluctuations in neuromuscular performance. Accordingly, while the intervention appears beneficial for explosive fitness overall, the evidence for a sequence-specific effect on standing long jump is not particularly strong.

By contrast, grip strength and vital capacity did not show significant group effects. These null findings are also understandable. Grip strength is a relatively specific indicator that may not be highly responsive to a lower-body-dominant school-based program centered on sprinting and bodyweight calisthenics.^25^ Likewise, changes in vital capacity may require either a longer intervention period or a more specific respiratory stimulus before clear between-group differences emerge. Thus, the absence of significant effects for these outcomes should not be interpreted as evidence that the intervention was ineffective overall, but rather that the training stimulus was more closely aligned with some physical capacities than others.

A significant group effect was also observed for resting heart rate, with both intervention groups showing lower adjusted post-intervention values than the control group. This finding is meaningful because resting heart rate is often viewed as a simple physiological marker related to cardiovascular adaptation and autonomic regulation.^29^ The reduction in resting heart rate in both training groups is consistent with the broader cardiorespiratory improvements observed in the present study and provides additional support for the cardiovascular benefits of this school-based program.^10^^,^^11^^,^^13^ However, systolic and DBP did not differ significantly among groups. This may reflect the relatively short duration of the intervention, the modest baseline variability in blood pressure, or the fact that blood pressure in adolescents can be influenced by multiple short-term behavioral and environmental factors that are difficult to fully control in a school setting.

Taken together, these secondary findings reinforce two broader points. First, the intervention was capable of improving several performance-related outcomes beyond the primary endpoint of VO_2_max. Second, the sequence effect remained limited across most of these measures. In practical terms, this suggests that teachers and practitioners may expect broad fitness benefits from incorporating both sprint-based and bodyweight resistance exercise into school sessions, even if the precise order is adapted to suit lesson organization. At the same time, the results also indicate that some outcomes, particularly more specific or less responsive physiological indicators, may require either a longer training period or a more targeted intervention strategy.

### Practical implications for school-based programming

One practical strength of the present study is that the intervention was delivered within a real school setting rather than under tightly controlled laboratory conditions. This is important because school-based obesity interventions are often constrained by limited class time, large group sizes, restricted equipment, and the need to fit within existing physical education schedules.^9^^,^^10^ Under these conditions, an intervention that is effective, simple to organize, and adaptable to routine teaching practice is likely to be more valuable than one that is theoretically optimal but difficult to implement.

The current findings suggest that both exercise sequences are feasible options for school-based programming.^11^^,^^18^ Across most outcomes, the two intervention groups showed similar benefits relative to the control group, indicating that a combined session including both repeated sprint and bodyweight resistance components can be effective even when the order is adjusted to meet practical teaching demands. This has direct relevance for physical education teachers. In many schools, one class may need to be divided into subgroups because of space limitations, teacher supervision demands, or the availability of equipment. In such situations, one subgroup can begin with sprint-based exercise while another starts with bodyweight resistance exercise, and the groups can then rotate. The present findings suggest that this kind of flexible organization is unlikely to substantially weaken the overall health-related benefits of the session.^21^

At the same time, the results also indicate that order may still matter when improving aerobic fitness is the primary objective. Because CRS showed a greater improvement in VO_2_max than CSR, teachers or practitioners who want to maximize cardiorespiratory gains may prefer to arrange the lesson so that the resistance-based calisthenics component precedes the sprint-based component. However, this should not be interpreted as meaning that CSR is ineffective. Rather, the practical message is that sequence can be optimized when a specific outcome is prioritized, while still allowing flexibility when broader health promotion and classroom manageability are the main concerns.

Another useful implication concerns intervention acceptability and scalability. The training format used in the present study relied on movements that are relatively easy to teach, do not require expensive equipment, and can be incorporated into ordinary school timetables.^9^ This makes the approach potentially transferable to a wide range of educational settings, including schools with limited resources. For adolescents with obesity, this is particularly relevant because participation barriers may already be high, and interventions that depend on specialized facilities are less likely to be sustained in routine practice.

Overall, the present study supports the idea that time-efficient, school-based high-intensity combined exercise can be used as a realistic strategy to improve health-related fitness and body composition in adolescents with obesity.^9^^,^^13^ From an implementation perspective, the most important priority may be to ensure that students participate regularly and complete both training components with adequate quality, rather than to impose a rigid sequence in every lesson. Exercise order can still be considered, but it appears to be a secondary programming decision rather than the main determinant of intervention success.

### Terminology and interpretation

An important issue in interpreting the present findings concerns the terminology used to describe the intervention. In classical exercise science literature, “concurrent training” usually refers to the combination of endurance exercise and resistance training, often in laboratory or athletic settings where the training modes are clearly separated and the resistance component involves conventional strength training with external load.^19^^,^^20^ The intervention in the present study differed from that model in several ways. The aerobic component consisted of repeated short sprint efforts, and the resistance component consisted of bodyweight calisthenics performed in a school-based physical education context. Accordingly, the present protocol should not be viewed as a direct equivalent of traditional laboratory-based concurrent training programs.^20^

This distinction matters because it affects how the results should be interpreted. The findings of the present study are better understood as evidence about the within-session order of two practical school-based high-intensity exercise components, rather than as a strict test of the classic “interference effect” framework.^19^ Although the idea of order-dependent adaptation remains relevant, the mechanisms discussed in traditional concurrent training research may not transfer directly to a protocol based on repeated sprints and bodyweight movements performed by adolescents in a classroom setting.^18^^,^^20^ For this reason, caution is needed when comparing the present results with studies using cycle ergometry, treadmill running, or externally loaded resistance training.

This perspective may also help explain why the order effect was selective rather than universal. If the present intervention is interpreted too narrowly through the lens of the conventional interference literature, there is a risk of expecting clear and systematic sequence effects across all outcomes. However, the school-based nature of the program, the developmental stage of the participants, and the mixed demands of repeated sprinting and calisthenics make the physiological context more complex. In this setting, it is perhaps more realistic to expect that exercise order will influence some outcomes, such as VO_2_max, while having only limited impact on many others.^18^^,^^19^^,^^30^^,^^31^

Therefore, the present study contributes less to a strict debate over whether one traditional concurrent training sequence is universally superior, and more to the practical question of how two high-intensity exercise components can be arranged within a school lesson for adolescents with obesity. Interpreted in this way, the findings become more directly relevant to physical education practice while also remaining consistent with the observed pattern of modest and outcome-specific order effects.

In conclusion, both exercise sequences produced beneficial effects on cardiorespiratory fitness, body composition, and several functional fitness outcomes in adolescents with obesity when delivered within a school-based setting. Overall, the influence of exercise order appeared to be limited, as both CRS and CSR showed broadly similar advantages over the control group across most outcomes. However, the order effect was not entirely absent. CRS resulted in a greater improvement in VO_2_max than CSR, suggesting that exercise sequence may still matter when cardiorespiratory fitness is the primary target of intervention.

These findings indicate that school-based programs combining repeated sprint exercise and bodyweight resistance exercise can be implemented as practical and time-efficient strategies for improving obesity-related health outcomes in adolescents. From an applied perspective, the overall training stimulus and consistent participation may be more important than strict control of exercise order for most outcomes. Nevertheless, when maximizing aerobic fitness is a priority, arranging the resistance component before the sprint component may be preferable.

### Limitations of the study

Several limitations should be acknowledged. First, VO_2_max was assessed using both direct measurement and estimation from the 20-m shuttle run test. Although the sensitivity analysis indicated that the main findings were not materially influenced by measurement method, this mixed approach introduced methodological heterogeneity. Second, maturation-related variables such as Tanner stage, maturity offset, sitting height, or bone age were not assessed. Because participants were adolescents undergoing normal growth, residual confounding by pubertal maturation may have affected the interpretation of bone-related outcomes. Third, dietary intake and physical activity outside the intervention sessions were only partially controlled. Although school lunches were standardized and breakfast guidance was provided, dinner intake, weekend meals, and free-living physical activity were not rigorously monitored, which may have influenced body composition and cardiovascular outcomes.

Additional methodological limitations should also be considered. The homogeneity of variance assumption was not satisfied for several outcomes, so findings for these variables should be interpreted cautiously. The 12-week intervention may also have been too short to capture longer-term adaptations, particularly for skeletal and cardiovascular indicators. Finally, although all measurements followed standardized procedures and were performed by trained assessors, no within-sample test-retest reliability analysis was conducted. Future studies with longer follow-up, more consistent physiological measurement procedures, maturation assessment, and better monitoring of diet and free-living activity are needed to clarify sequence-related responses in adolescents with obesity.

## Resource availability

### Lead contact

Further information and requests for resources should be directed to and will be fulfilled by the Lead Contact, Meng Cao (caomengsus@163.com).

### Materials availability

This study did not generate new unique reagents or materials.

### Data and code availability

• The participant-level data supporting the findings of this study are not publicly deposited because the study involved minors and the data are subject to ethical and privacy restrictions. De-identified data may be made available by the lead contact upon reasonable request and subject to approval by the relevant ethics committee.
• This study does not report original code. All statistical analyses were conducted using IBM SPSS Statistics 25.0, as described in the STAR Methods.
• Any additional information required to reanalyze the data reported in this study is available from the lead contact upon request.

## Acknowledgments

We are grateful to the participating students and their families, the partnering schools and physical education teachers for their collaboration during testing and training sessions. We also thank the pediatric consultants and school administrators for facilitation of screening and safety oversight, and the research assistants who supported data collection and quality control. This work was supported by the Futian Healthcare Research Project (no. FTWS109) and the Futian Healthcare Research Project (no. FTWS2025074), Shenzhen Futian Third People’s Hospital, Shenzhen, China.

## Author contributions

Y.G.: conceptualization, methodology, investigation, formal analysis, data curation, visualization, and writing – original draft; C.D.: methodology and writing – review and editing; J.K.: investigation and data curation; X.W.: investigation and resources; T.Z.: data validation and writing – review and editing; M.C.: conceptualization, methodology, supervision, project administration, writing – review and editing, and correspondence; Y.X.: resources, clinical coordination, participant recruitment and testing coordination, data validation, funding acquisition, writing – review and editing, and correspondence. All authors read and approved the final manuscript and agree to be accountable for all aspects of the work.

## Declaration of interests

The authors declare no competing interests.

## STAR★Methods

### Key resources table

**Table:** 

REAGENT or RESOURCE	SOURCE	IDENTIFIER
**Deposited data**		

Clinical trial registration	Chinese Clinical Trial Registry	ChiCTR2500103429
Dataset generated in this study	This paper	Available from the Lead Contact upon reasonable request and subject to ethical and privacy restrictions
WHO 2007 growth reference	de Onis et al.^32^	https://doi.org/10.2471/BLT.07.043497

**Software and algorithms**		

IBM SPSS Statistics 25.0	IBM Corp.	RRID: SCR_002865; https://www.ibm.com/products/spss-statistics
G∗Power 3.1	Heinrich Heine University Düsseldorf	RRID: SCR_013726; https://www.psychologie.hhu.de/arbeitsgruppen/allgemeine-psychologie-und-arbeitspsychologie/gpower

**Other**		

COSMED K5 wearable metabolic system	COSMED, Rome, Italy	Model: K5
20-m shuttle run test protocol	Léger et al.^33^	https://doi.org/10.1080/02640418808729800; Léger et al.^33^
VO2max estimation equation for the 20-m shuttle run test	Mahar et al.^34^	https://doi.org/10.1016/j.amepre.2011.07.008
Dual-energy X-ray absorptiometry system for body composition	GE Healthcare, USA	Model: Lunar Prodigy
Dual-energy X-ray absorptiometry system for bone assessment	GE Healthcare, Madison, WI, USA	Model: iDXA
Automated blood pressure monitor	Omron Healthcare, Osaka, Japan	Model: HEM-1020
Heart rate monitoring system	Polar Electro Oy, Finland	Polar Team Pro
Hand dynamometer	Lafayette Instrument Company, Lafayette, IN, USA	Model: Jamar 5030J1

### Experimental model and study participant details

#### Study design and setting

This was a 12-week, three-arm, parallel-group randomized controlled trial conducted in accordance with the CONSORT guidelines.^35^ The protocol was approved by the medical ethics committee of the Department of Medicine of Shenzhen University (PN-202400005), registered on the Chinese Clinical Trial Registry (ChiCTR2500103429), and conducted according to the Declaration of Helsinki. Written informed consent was obtained from legal guardians, with assent provided by all participants.

#### Participants

Adolescents were eligible if they were 11–13 years old; Tanner stage 1–3 as assessed by a pediatrician; had BMI at or above the 95th percentile for sex and age; had no contraindications to physical activity; reported less than 2 h week^−1^ of habitual physical activity (International Physical Activity Questionnaire, short form)^32^; and were free from any condition or medication that could interfere with the protocol (e.g., cardiac, orthopedic, neuromuscular, or neurological disorders).^36^

Exclusion criteria were a history of major cardiovascular, metabolic, or neurological disease; current use of medication affecting exercise responses or metabolism; metal or electrical prostheses; participation in another weight-loss or exercise program; inability to understand or follow the protocol; or being deemed unsuitable by investigators.

#### Randomization and group allocation

A total of 100 adolescents with obesity were screened, of whom 60 met the eligibility criteria and were randomized (1:1:1) using a computer-generated sequence in SPSS software (version 25.0, IBM Corp., Armonk, NY, USA) to one of three groups: a non-exercising control group (CON), bodyweight resistance exercise followed by repeated sprint exercise (CRS), or repeated sprint exercise followed by bodyweight resistance exercise (CSR). Allocation was concealed from outcome assessors and data analysts.

#### Sample size estimation

Sample size was estimated *a priori* using G∗Power software (version 3.1, University of Düsseldorf, Germany).^37^ The calculation was based on the primary outcome, VO_2_max, for a three-group, two-time-point repeated-measures design. An alpha level of 0.05 and a statistical power of 0.80 were specified. The assumed effect size was set at f = 0.25 (moderate), based on previous studies reporting exercise-related improvements in cardiorespiratory fitness in children and adolescents with obesity. On this basis, the minimum required sample size was estimated to be 48 participants in total. Considering a potential dropout rate of approximately 20%–25%, the target sample size was increased to 60 participants (20 per group).^13^^,^^38^

### Method details

#### General assessment procedures

Baseline (week 0) and post-intervention (within 3 days after the 12-week program) assessments were conducted in standardized laboratory and field settings following the testing sequence illustrated in the main manuscript. To minimize acute exercise and dietary effects, participants avoided vigorous physical activity and caffeine or sugar-sweetened beverages for at least 12 h and consumed a standardized breakfast according to study guidance. Pre- and post-tests were scheduled at the same time of day (10:00–12:00) for each participant to reduce diurnal variation. All measurements were performed by trained assessors blinded to group allocation, and data analysts were also blinded.

All outcome assessments were conducted using standardized procedures by trained assessors. Reliability evidence for these measurements was based on established protocols and prior literature, but no formal within-sample test–retest analysis was performed in the present study.

#### Dietary control and standardization of school meals

Dietary intake was not manipulated as a primary component of the intervention. However, basic dietary control procedures were implemented to minimize large between-group differences and within-subject changes in energy intake over the 12-week study period. All participants attended the same public school and consumed a standardized school lunch prepared by the school canteen on each school day. The lunch menu was planned according to local nutritional guidelines for adolescents and was identical across classes and study groups. Although individual portion sizes could not be strictly controlled, all participants had access to the same dishes and side items at lunchtime throughout the intervention.

To reduce variability in pre-exercise energy intake, participants were instructed to consume breakfast before 07:30 on school days, consisting of staple foods (e.g., rice porridge, steamed buns, or bread) and milk or soy milk, while avoiding sugar-sweetened beverages and deep-fried snacks. Parents and guardians received written guidance and were asked to help their children follow these breakfast instructions during the intervention. Dinner and weekend meals were not controlled by the research team. Participants and their families were instructed to maintain habitual eating patterns and to avoid intentional weight-loss diets, marked overfeeding, or major dietary changes during the study. No structured nutritional education sessions were delivered to any group.

#### Intervention procedures

Participants in both exercise groups completed supervised sessions three times per week for 12 weeks on non-consecutive days. Each session comprised warm-up, main exercise, and cool-down components, and lasted approximately 20 min. The only difference between the two intervention groups was the order of the two main exercise components.

#### Bodyweight resistance exercise

The bodyweight resistance component consisted of a 10-min, six-station circuit-based exercise sequence performed using fixed work and transition intervals at high perceived exertion, followed by 1-min seated rest. The six exercises were fast-feet with punches, jumping jacks, alternating knee-to-elbow (standing), floor-touch squats, half-squat to front kicks, and under-thigh-clap hops.

#### Repeated sprint exercise

The repeated sprint component consisted of repeated all-out 20-m sprints from a standing start, performed according to a fixed work–recovery structure rather than heart-rate targets.

#### Exercise order

The CRS group performed bodyweight resistance exercise followed by repeated sprint exercise, whereas the CSR group performed repeated sprint exercise followed by bodyweight resistance exercise (see figure below).

**Figure:**
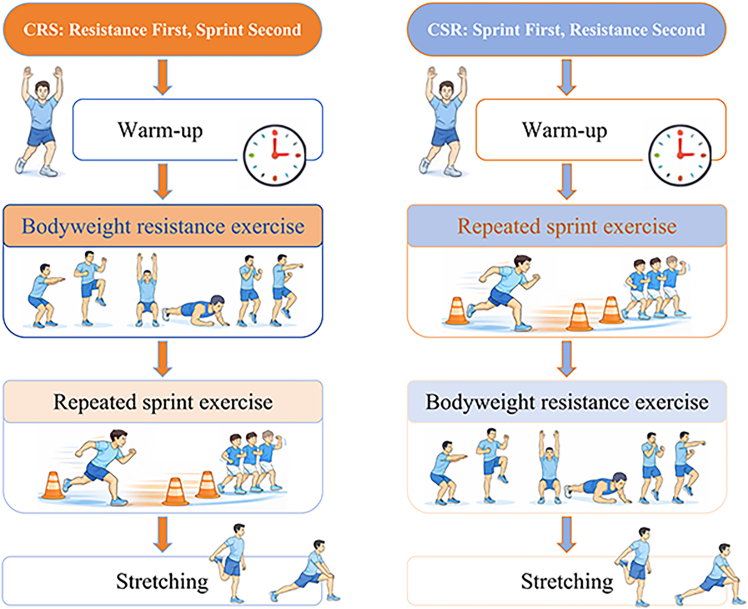
Within-session exercise order in the CRS and CSR groups The CRS group performed bodyweight resistance exercise followed by repeated sprint exercise, whereas the CSR group performed repeated sprint exercise followed by bodyweight resistance exercise. Both groups completed the same exercise components, frequency, and overall session duration.

#### Monitoring and fidelity

All sessions were supervised by trained research staff. Heart rate was recorded for monitoring and safety purposes rather than as a target for exercise prescription. Attendance, rating of perceived exertion (RPE), and any adverse events or participant discomfort were recorded throughout the intervention.

#### Anthropometry and body composition

Height was measured to the nearest 0.1 cm using a wall-mounted stadiometer, and body mass to 0.1 kg using a calibrated digital scale, with participants barefoot and in light clothing. Waist and hip circumferences were measured with a non-elastic tape according to standardized landmarks, and waist-to-hip ratio (WHR) was calculated. Whole-body body fat percentage (BF%) and fat-free mass (FFM) were obtained from dual-energy X-ray absorptiometry (DXA; Lunar Prodigy, GE Healthcare, USA).^39^ Age- and sex-adjusted BMI-z scores were derived from the 2007 WHO growth reference using the LMS method.^32^

(Equation 1) $$BMI\;z-score=\frac{{\left(BMI/M\right)}^{L}-\;1}{\left(L\times S\right)}\;if\;L\neq 0$$

(Equation 2) $$BMI\;z-score=\frac{\ln\left(BMI/M\right)}{S}\;if\;L=0$$

#### Cardiorespiratory fitness

Cardiorespiratory fitness was assessed as maximal oxygen uptake (VO_2_max). Because of limited availability of the portable metabolic system and constraints on weekend testing time, VO_2_max could not be directly measured in all participants. Therefore, approximately half of the participants in each group underwent a maximal graded exercise test with breath-by-breath gas analysis using a portable metabolic cart (K5, COSMED, Rome, Italy). VO_2_max was defined as the highest 30-s averaged value achieved during the test according to standard criteria for maximal effort.^40^

Direct VO_2_max measurement using the COSMED K5 was performed in the first 10 participants tested within each group (CON: 10/20; CRS: 10/19; CSR: 10/20), whereas the remaining participants were assigned estimated VO_2_max values derived from the 20-m shuttle run test. This resulted in a near-balanced distribution of measurement methods across groups (30 direct measurements and 29 estimated values in total).

For the remaining participants, VO_2_max was estimated from performance on the 20-m shuttle run test (20-m SRT) following the Léger protocol.^33^ After a 5-min jog and dynamic drills, participants ran back and forth between two 20-m lines in time with audio beeps, starting at 8.5 km h^−1^ and increasing by 0.5 km h^−1^ each minute. The test ended at volitional exhaustion or when a participant twice failed to reach the line on the beep. Laps and the highest fully completed stage (plus partial-stage shuttles) were recorded by two assessors, and VO_2_max (mL·kg^−1^·min^−1^) was estimated using the Mahar youth equation.^34^

(Equation 3) $${\text{VO}}_{2}\;\max=41.76799+\left(0.49261\times \text{laps}\right)-\;\left(0.00290\times \text{lap}s^{2}\right)\;-\;\left(0.61613\times \text{BMI}\right)+\left(0.34787\times \text{gender}\times \text{age}\right)$$

Resting systolic and diastolic blood pressure (SBP/DBP) and resting heart rate (RHR) were measured after 10 min of seated rest using an automated sphygmomanometer (Omron HEM-1020, Osaka, Japan). Heart rate was continuously monitored during testing (Polar Team Pro, Polar Electro, Finland).

#### Bone health

Whole-body areal bone mineral density (BMD), bone mineral content (BMC), BMD Z-scores, and whole-body FFM were assessed using dual-energy X-ray absorptiometry (DXA; iDXA, GE Healthcare, Madison, WI, USA). To standardize hydration and loading status, participants attended morning visits, avoided vigorous exercise for at least 12 h, fasted for 2–3 h (water allowed), voided within 30 min before scanning, and removed all metal objects; baseline and follow-up scans were time-of-day matched on the same device with the same technician.^39^ The scanner was calibrated daily with the manufacturer’s phantom. Participants were positioned supine according to the pediatric whole-body protocol. Two total-body-less-head scans with full repositioning were acquired and averaged, and scans affected by motion or artifact were repeated. Regions of interest were reviewed and adjusted as needed. Z-scores were based on the manufacturer’s age- and sex-specific pediatric reference. Effective radiation dose was less than 10 μSv per scan.

Each whole-body DXA scan required approximately 5 min of acquisition time. Including participant positioning, removal of metallic objects, and immediate quality-control review, the total time per participant was approximately 8–10 min. Baseline whole-body DXA measurements for all 60 participants were completed across four testing days, and follow-up assessments were organized in the same manner to ensure time-of-day matching.

#### Functional fitness and physiological outcomes

All physical-fitness tests were administered indoors on non-slip flooring under standardized environmental conditions (25°C–27°C). Participants wore athletic shoes and completed a structured 5-min dynamic warm-up (jogging, leg swings, arm circles, and submaximal jumps) before testing. Tests were performed in a fixed order to minimize fatigue carryover. All assessments were conducted by trained examiners following the national student physical-fitness test protocols.

Handgrip strength was measured using a calibrated hydraulic hand dynamometer (Jamar 5030J1, Lafayette Instrument Company, Lafayette, IN, USA). Two trials were performed for each hand alternately, with at least 30 s rest between trials, and the highest value obtained from either hand was recorded as maximal voluntary contraction (kg).

Standing long jump was used to assess lower-limb power. Participants stood behind the take-off line and jumped forward as far as possible using a countermovement and arm swing. Two attempts were allowed, and the longest distance was recorded to the nearest centimeter.

Abdominal endurance was evaluated using a 1-min sit-up test. Participants lay supine with the knees flexed at approximately 90°, feet secured, and hands crossed over the chest. A repetition was counted when the elbows touched the thighs and the shoulders returned to the mat. The total number of valid repetitions completed in 60 s was recorded.

Motor coordination and speed were assessed using a 1-min rope-skipping test. Participants used a standardized rope length and were instructed to skip continuously for 60 s at their maximal sustainable pace. The examiner counted successful jumps and subtracted obvious misses from the total to obtain the final score.

### Quantification and statistical analysis

All statistical analyses were performed using IBM SPSS Statistics 25.0 (IBM Corp., Armonk, NY, USA). Continuous variables are presented as mean ± standard deviation (SD), unless otherwise stated, and categorical variables are presented as counts. Statistical significance was set at a two-sided α level of 0.05.

Baseline differences among groups were assessed using one-way analysis of variance (ANOVA) for continuous variables and the chi-square test for categorical variables. For the main outcome analyses, analysis of covariance (ANCOVA) was used to compare post-intervention outcomes among groups, with group as the fixed factor and the corresponding baseline value of each outcome entered as a covariate. Adjusted post-intervention means with 95% confidence intervals (CIs) were estimated for each group. When a significant group effect was detected, Bonferroni-adjusted pairwise comparisons were performed. Effect sizes for group effects were expressed as partial eta squared (partial η^2^).

For descriptive purposes, pre- and post-intervention values are presented as mean ± SD, and change scores are reported as mean differences with 95% CIs. Pairwise effect sizes were additionally quantified using Hedges’ g based on change scores and are presented in the Supplementary Material.

Because VO_2_max was assessed using two methods (direct measurement or estimation from the 20-m shuttle run test), a sensitivity analysis was conducted for VO_2_max by repeating the ANCOVA with measurement method included as an additional fixed factor. In this model, baseline VO_2_max was retained as a covariate, and the main effects of group and measurement method, as well as the group × method interaction, were examined.

The assumptions of ANCOVA, including homogeneity of variance, were checked before inference. Outcomes for which the homogeneity of variance assumption was violated were interpreted with caution.

## Supplemental information

Document S1. Tables S1 and S2

Document S2. CONSORT checklist
