# Supplementary material for: Exercise order in school-based concurrent training for adolescents with obesity
Source: iScience. 2026 Jul 6;29(7):116136. doi: 10.1016/j.isci.2026.116136 (PMC13355818; doi:10.1016/j.isci.2026.116136)
Supplement: Document S1. Tables S1 and S2 [file mmc1.pdf]

iScience, Volume *29*

Supplemental information

Exercise order in school-based concurrent training for adolescents with obesity

Yuhang Gao, Chunyan Dai, Jiahui Ke, Xiaodong Wang, Ti Zhang, Meng Cao, and Yan Xie

Table S1. Pairwise effect sizes (Hedges' g) for change scores across outcomes

Outcome	CRS vs CON	CSR vs CON	CRS vs CSR
Weight	0.563	-1.942	2.836
BMI-z	0.283	-1.777	1.934
BMI	-2.525	-1.593	-0.996
Waist circumference	-2.558	-2.165	-0.225
Hip circumference	-0.582	-0.029	-1.014
WHR	-0.962	-1.284	0.427
Fat-free mass	0.966	1.449	0.208
Standing long jump	0.832	1.255	-0.624
Rope skipping	0.614	0.93	-0.197
Sit-ups	0.713	1.019	-0.207
Grip strength	0.206	0.079	0.075
Vital capacity	0.637	0.587	0.078
Systolic blood pressure	0.108	-0.235	0.293
Diastolic blood pressure	0.028	-0.488	0.473
Resting heart rate	-1.295	-1.01	-0.358
20-m shuttle run test	2.069	1.938	0.534
VO_2max	3.206	2.442	0.772
BMD	-0.66	-0.935	0.204
Z-score	0.145	0	0.151
BMC	0.052	0.191	-0.152
BF%	-2.01	-2.592	-0.263

Table S2. Sensitivity analysis for VO_2max after additional adjustment for measurement method

Outcome	Sensitivity model	Group effect F	p	Method effect p	Group × Method p
VO_2max	Adjusted for baseline VO_2max and measurement method	43.84	<0.001	0.275	0.609
